# Crystallization and Optical Behaviour of Nanocomposite Sol-Gel TiO_2_:Ag Films

**DOI:** 10.3390/molecules29215156

**Published:** 2024-10-31

**Authors:** Tatyana Ivanova, Antoaneta Harizanova, Tatyana Koutzarova, Raphael Closset

**Affiliations:** 1 Central Laboratory of Solar Energy and New Energy Sources, Bulgarian Academy of Sciences, Tzarigradsko Chaussee 72, 1784 Sofia, Bulgaria; tativan@phys.bas.bg; 2 Institute of Electronics, Bulgarian Academy of Sciences, Tzarigradsko Chaussee 72, 1784 Sofia, Bulgaria; tanya@ie.bas.bg; 3 GREENMAT, Institute of Chemistry B6, University of Liege, B6a, Quartier Agora, Allee du Six Août, 13, 4000 Liège, Belgium; raphael.closset@uliege.be

**Keywords:** sol-gel deposition, silver nanoparticles, TiO_2_ films

## Abstract

Sol-gel spin coating method was employed for depositing TiO_2_ and Ag-doped TiO_2_ films. The effects of Ag doping and the annealing temperatures (300–600 °C) were studied with respect to their structural, morphological, vibrational, and optical properties. Field Emission Scanning Electron microscopy (FESEM) investigation exhibited the grained, compact structures of TiO_2_-based films. Ag incorporation resulted in a rougher film surface. X-ray diffraction (XRD) results confirmed the formation of Ag nanoparticles and AgO phase, along with anatase and rutile TiO_2_, strongly depending on Ag concentration and technological conditions. AgO fraction diminished after high temperature annealing above 500 °C. The vibrational properties were characterized by Fourier Transform Infrared (FTIR) spectroscopy. It was found that silver presence induced changes in IR bands of TiO_2_ films. UV–VIS spectroscopy revealed that the embedment of Ag NPs in titania matrix resulted in higher absorbance across the visible spectral range due to local surface plasmon resonance (LSPR). Ag doping reduced the optical band gap of sol-gel TiO_2_ films. The optical and plasmonic modifications of TiO_2_:Ag thin films by the number of layers and different technological conditions (thermal and UV treatment) are discussed.

## 1. Introduction

Titanium dioxide (TiO_2_) is a wide-band gap semiconductor widely studied owing to its unique optical properties (high optical transparency in visible spectral range, high refractive index above 2.4) [1], high chemical and thermal stability [2], large surface area, non-toxicity, and cost effectiveness compared to other semiconductors [1]. These characteristics turn TiO_2_ and TiO_2_-based structures multifunctional materials, suitable for a huge number of applications in the fields of catalysis and photocatalysis, gas sensors, energy storage, self-cleaning devices, optical and corrosion protective coatings [2,3], optical waveguides, ultraviolet (UV) light shielding, photovoltaics [4], anti-reflection coatings, multilayer optical coatings, the fields of photocatalytic water decomposition [5], photocatalytic organic degradation, and artificial photosynthesis [6]. In particular, the research on TiO_2_ usage in water pollution degradation has experienced a notable increase due to its non-toxicity, non-polluting nature, high chemical stability, and low cost [7]. TiO_2_ exists in three main crystallographic forms: anatase, rutile, and brookite as each crystal phase manifests different optical properties (band gap, refractive index), surface state, and physical properties [8]. In addition to the three mentioned structures, there are a number of metastable, less-studied, and polymorphic forms of TiO_2_, including TiO_2_(B), hollandite-like TiO_2_, and ramsdellite-like TiO_2_, as well as high-pressure forms [9]. There are also Magnéli phases, i.e., titanium suboxides. The fundamental difference in these polymorphs is related to the connectivity, the volume, and the shape of TiO_6_ octahedra, as well as the distortion of the individual TiO_6_ octahedron [10]. The crystal structures of rutile and anatase belong to the tetragonal space groups *P*4_2_/mnm a = b = 4.593 Å, c = 2.959 Å, and *P*4_1_/amd, a = b = 3.785 Å, c = 9.514 Å, respectively [10]. Brookite-type TiO_2_ exhibits orthorhombic geometry with space group P_bca_ [8], a = b = 5.436 Å, c = 9.166 Å. The unit cell of tetragonal anatase contains four TiO_2_ units (12 atoms), while the unit cell of tetragonal rutile contains two TiO_2_ units (6 atoms), and the unit cell of orthorhombic brookite contains eight TiO_2_ units (24 atoms) [11]. Rutile crystal phase is supposed as the most thermodynamically stable phase. Anatase titania exists in tetragonal geometry with strongly distorted TiO_6_ octahedra. Both crystal structures consist of TiO_6_ octahedra, sharing four edges in anatase and two in rutile [8]. Brookite TiO_2_ Brookite is the least stable phase among the three due to the orthorhombic structure and it is found to be difficult to synthesize [11].

As a polymorphous compound, the crystallographic structure, titania phases, morphology and physical properties are essential for the applications mentioned above [12]. For example, anatase TiO_2_ is reported to possess higher photocatalytic activity compared to rutile, although the optical band gap is larger (3.2 eV for anatase vs. 3.0 eV for rutile) [13]. It is worth mentioning that the material with the phase mixture of anatase and rutile polymorphs is reported to have synergistic effects, showing increased photocatalytic activity compared to pure phases [14].

The study of the factors affecting the phase transformation of anatase/rutile (A–R) is very important, especially for devices based on titanium dioxide [15]. The appearance of the phase transition can alter the properties and performance of the devices. Therefore, the knowledge of TiO_2_ polymorphs and the phase transition kinetics is essential to the ability to obtain single-phase or multiphase microstructures [12]. The formation of anatase and rutile TiO_2_ depends on the synthesis method and the deposition parameters (deposition or annealing temperature, time). The reported transition temperatures vary in a broad range of 400–1200 °C [16,17], determined by the product preparation technique, temperature, particle sizes, atmosphere, impurities and dopants morphology, etc. The anatase to rutile transformation is reconstructive, involving the breaking and reforming of bonds [17]. This is in contrast to a displacive transformation, in which the original bonds are distorted but retained. The phase transition temperature in TiO_2_ thin films can be altered by adding a dopant. The presence of impurities or dopants may imply the lattice distortions thus affecting the degree of crystallinity. Depending on the type of the dopant, the anatase/rutile transition temperature can be changed [17].

Silver (Ag) is theoretically predicted as a cation promoter for the anatase-rutile transition in titanium dioxide [18,19]. Combination of noble metal nanoparticles (Ag, Au, Pd, Pt) within TiO_2_ matrix significantly tunes its physical and chemical properties [20]. Plasmonic nanoparticles (NPs) and nanostructures, consisted of metal oxides with embedded NPs have great potential in photovoltaics, nano-imaging, bio-sensing, photoelectric sensors, and light engineering metamaterials [21]. Ag possesses a great advantage of lower toxicity, lower cost, high electrical conductivity, biological properties, including higher antibacterial property compared to Au and Pt, useful for wastewater treatment [22]. The other intriguing property of Ag NPs incorporated in TiO_2_ is the enhancement of the absorbing light at specific wavelengths across the visible part of the electromagnetic spectrum due to localized surface plasmon resonance (LSPR). LSPR refers to the collective oscillation of electrons on the metallic NPs excited by the incident photons at the resonant frequency. Metallic NPs embedded in dielectric host matrix such as TiO_2_ have been extensively adapted in a wide range of applications, such as in solar cells, sensors, energy harvesting, enhanced photocatalysis and biomedical devices [23]. The other usage and great potential of TiO_2_:Ag nanostructures have been shown in the field of nanobiotechnology for antibacterial applications, sensitive surface-enhanced Raman scattering (SERS) sensor, in photovoltaic cells [24], etc.

Our previous paper [25] gave the preliminary results for the structural and optical properties of TiO_2_:Ag films, obtained from sol solutions with AgNO_3_ concentrations of 1 and 0.5 wt%, Sol A and Sol B after annealing at 600 °C. These results were compared to TiO_2_:Ag films, obtained from these sols, but the films were UV treated between layer deposition and finally treated at 600 °C.

This work presents a new systematic investigation of sol-gel deposited silver-doped TiO_2_ films. In this study, two sets of TiO_2_:Ag nanocomposite films were prepared. The first group contained TiO_2_:Ag films, obtained from sols with three Ag precursor concentrations, and the second set consisted of TiO_2_:Ag films, deposited from preliminary treated sol solution with UV radiation. The samples were thermally treated at the temperatures from 300 to 600 °C. The aim is to investigate the influence of silver amount and thermal annealings on the structural, morphological, and optical properties of TiO_2_:Ag nanocomposite thin films. To correlate the optical properties of the films with the changes promoted by these two factors: Ag concentration and high temperature annealing.

## 2. Results and Discussion

### 2.1. FESEM Observation

**Figure 1:**
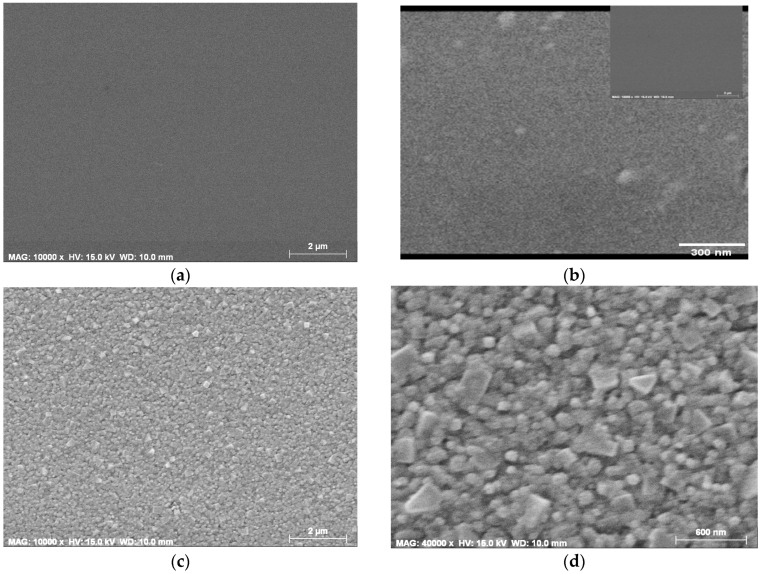

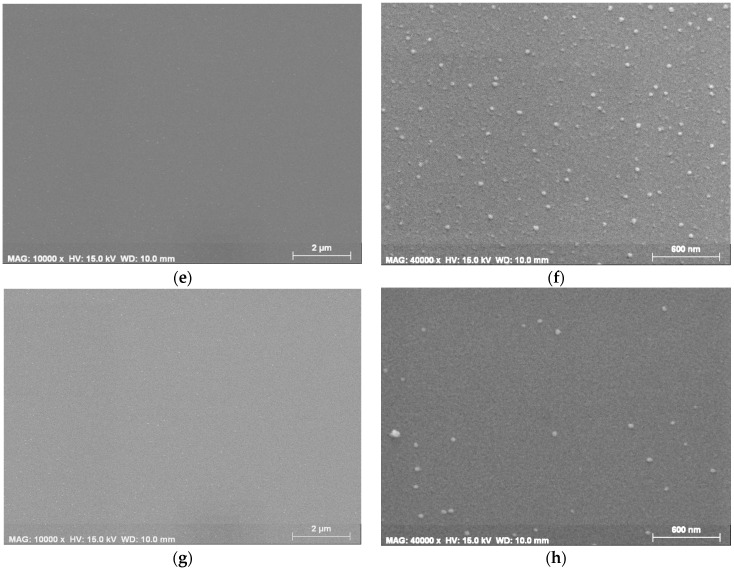
FESEM micrographs (magnification of 40,000×) of sol-gel films as (**a**,**b**) undoped TiO_2_ (with magnification 10,000× and 80,000×, respectively) and TiO_2_:Ag films, deposited from Sol A; (**c**,**d**) with magnification 10,000× and 40,000×, respectively; (**e**,**f**) film from Sol B (with magnification 10,000× and 40,000×, respectively), and (**g**,**h**) Sol C (with magnification 10,000× and 40,000×), respectively. The films were treated at 600 °C.

FESEM technique was applied to investigate the film morphology of undoped TiO_2_ and Ag embedded TiO_2_ films on Si substrates and thermally treated at 600 °C for 1 h. The FESEM micrograph (see Figure 1a) reveals that TiO_2_ film possesses a very smooth and uniform surface with tiny grainular structure.

It is seen that the film morphology is compact and continuous. The higher magnification (80,000×) shows the uniform, smooth film with some greater spherical grains with different sizes (from 43 to 50 nm), irregularly distributed on the surface.

Figure 1b presents the TiO_2_ film, incorporated with the highest content of Ag nanoparticles (Sol A, 1% wt AgNO_3_), annealed at 600 °C. The film surface is grained structured with a rougher surface compared to pure TiO_2_ film. The higher magnification of SEM image shows that the smaller particles are spherical and the greater ones are in irregular shapes. The sizes of the smaller grains are varied from 40 to 80 nm, and the bigger grains range from 200 to 320 nm in length. The structure is dense and the grains are clanged close to each other. TiO_2_:Ag film (Sol B, 0.5% wt AgNO_3_) has an uniform surface with tiny particles (Figure 1c). There can be also observed the formation of the spherical grains. The larger spherical particles are almost regularly spreaded over the film surface. The sizes of these particles range from 15 to 40 nm. TiO_2_:Ag film, deposited from Sol C (Figure 1c), with the lowest Ag concentration, (0.1% wt AgNO_3_), shows a very uniform surface morphology. The structure consists of small sized grains, with the smooth and dense film surface. The larger spherical particles are rarely distributed, with sizes ranging from 16 to 45 nm. The morphology is even smoother, with the larger particles seldomly spread on the film surface.

TiO_2_ and TiO_2_:Ag films exhibit rather smooth, grainular compact structures. It is evident from FESEM migrographs that the roughness, the average sizes of the grains change with Ag incorporation. The film morphology is gradually modified to a rougher film surface with increasing the silver concentration. The highest Ag doping results in rough film morphology with grains with different sizes and shapes. XRD study as presented below reveal that TiO_2_:Ag films possessed multiphase crystal phases strongly dependent on the initial Ag concentration and the annealing procedures.

### 2.2. XRD Study

#### 2.2.1. XRD Analysis—Effect of the Silver Concentration

The crystallization evolution with annealing temperature of the sol-gel TiO_2_ and TiO_2_:Ag films was studied by the XRD technique. The impact of the silver concentrations and t UV sol treatment was monitored.

**Figure 2:**
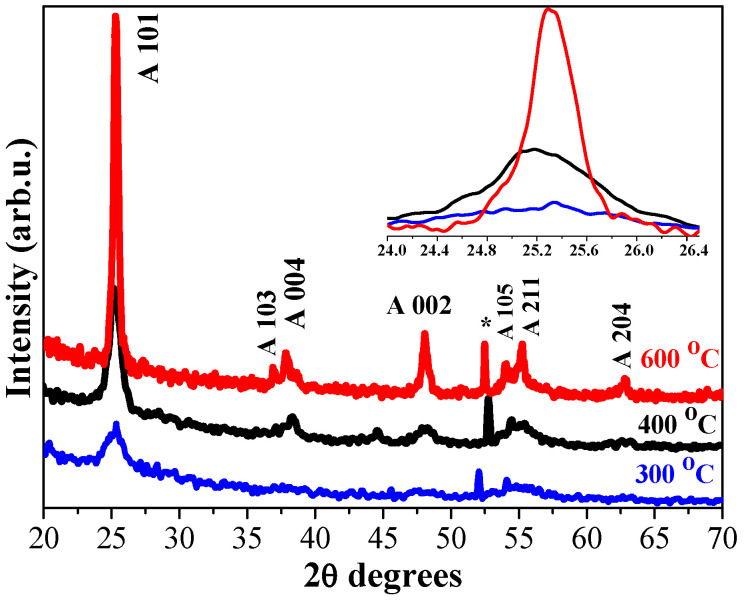
XRD patterns of TiO_2_ films, annealed at 300, 400, and 600 °C. The used substrate is Si wafer. The embedded figure presents a closed look at the strongest XRD line. The asterisk marks the line due to Si substrate.

Figure 2 displays XRD patterns of undoped TiO_2_ films, annealed at the temperatures from 300 to 600 °C. The films are found to polycrystalline. All the detected peaks match well with the anatase phase of TiO_2_ (PDF card 00-004-0477).

It is established that the film crystallization begins even at the lowest annealing temperature of 300 °C (Figure 2, blue curve), where wide XRD lines with low intensities appeared. With increasing the annealing temperature, the crystallization improves. The clear, sharp XRD features are seen for the sample, subjected to the highest annealing (600 °C). The well defined peak at 2θ = 25.32 degrees corresponds to the strongest line of anatase phase, indexed as 101 plane. The calculated crystallize sizes of anatase TiO_2_, derived from Scherrer’s equation (considering the diffraction plane 101), vary from 6.5 to 20 nm with increasing the annealing temperatures (from 300 to 600 °C).

**Figure 3:**
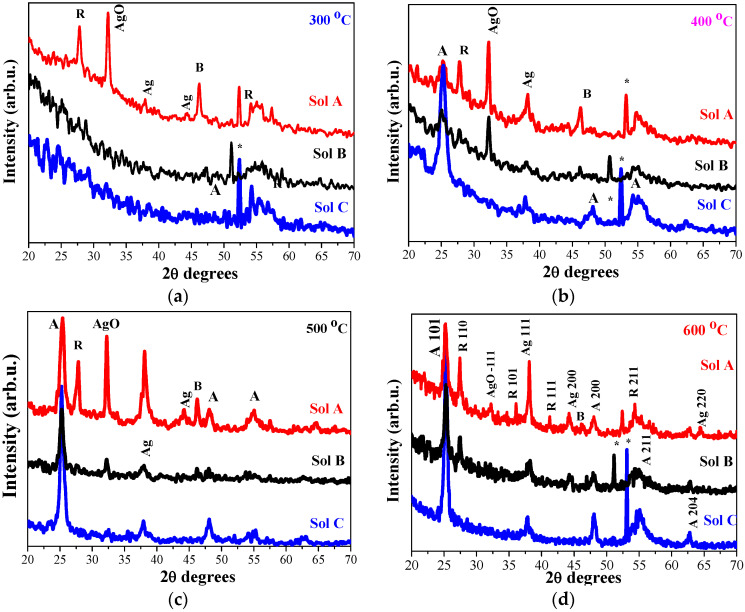
XRD patterns of sol-gel TiO_2_:Ag films, obtained from Sol A, Sol B, and Sol C, where (**a**) presents TiO_2_:Ag films, annealed at 300 °C; (**b**) at 400 °C; (**c**) at 500 °C; and (**d**) at 600 °C. Anatase phase is marked as **A**, rutile—**R**, brookite—**B**, asteriks * presents peaks due to Si substrate, respectively.

The inclusion of Ag NPs within the titanium oxide matrix modifies the film crystallization. Figure 3 shows the comparison of XRD patterns of the first group of TiO_2_:Ag films as a function of the annealing temperatures (300–600 °C). The films were deposited from Sol A, Sol B, and Sol C. The structural analysis affirms the significant influence of the the silver concentration on the film crystallization and the formation of the different crystal phases. The lowest annealing temperature (300 °C, Figure 3a) induces crystallization mainly in undoped TiO_2_ and in TiO_2_:Ag film, obtained from Sol A (the highest Ag addition). TiO_2_:Ag films, deposited from Sol B and Sol C manifest XRD patterns with no well-defined diffraction peaks (excepting the line assigned to Si substrate), which suggests a predominantly amorphous film structure. TiO_2_:Ag (Sol A) film possesses a more complicated structure, revealing XRD peaks assigned to different TiO_2_ crystal phases, to metallic Ag and to silver oxide. Table 1 presents the estimated crystallite sizes for TiO_2_ and TiO_2_:Ag films, annealed at 300–600 °C.

The weak line at 38.03 degrees is due to metallic Ag (PDF card 00-004-0783). The XRD line at 32.2 degrees can be referred to AgO phase (PDF card 00-043-1038). There are two weak lines that can be most probably related to brookite TiO_2_ (PDF card 01-071-4943). The appearence of XRD line at 27.6 degrees is attributed to rutile TiO_2_ phase (JCPDS No. 21-1276). The annealing temperature of 300 °C is actually very low for promoting the appearance of rutile phase. It is proposed that Ag insertion provokes the formation of the rutile phase. Previous study [26] reveals that the sol-gel TiO_2_ films crystallized in rutile phase after high temperature treatment at 800 °C.

All the studied films crystallized after annealing at 400 °C (Figure 3b). Sol A nanocomposite films show the coexistence of anatase, rutile, and brookite phases along with AgO and metallic cubic Ag. The corresponding crystallite sizes are estimated to be 10.1 nm (101, anatase), 20.5 nm (110, rutile), 24.8 nm (−111, AgO), and 21.8 nm for metallic Ag (111, cubic). XRD pattern of TiO_2_:Ag (Sol B) film indicates a polycrystalline structure with anatase phase (crystallite size of 9.2 nm), AgO phase (23.9 nm crystallite size). The rutile phase is also present with a broad line. The presence of silver (Ag^0^) with a broad weak line at 38.03 degrees is also detected. Ag-doped TiO_2_ film, obtained from Sol C, is starting to crystallize. The well-expressed lines are corresponding to the anatase phase (with a crystallite size of 9.9 nm, (101)). There is one line assigned to cubic Ag (111).

XRD patterns of 500 °C annealed films show interesting features (see Figure 3c). It seems that the rutile phase is suppressed in the Sol A film structure. XRD features related to the anatase phase are well defined and intense. The crystallite size of anatase is 12.3 nm. Ag^0^ phase is proved by the appearence of its two strongest peaks at 38.1 and 44.3 degrees. The crystallite size is 11.4 nm, estimated from 111 plane. TiO_2_:Ag (Sol B) film crystallizes in predominantly anatase phase (crystallite size of 15.6 nm). A weak diffraction feature (27.6 degrees) is assigned to rutile phase. The silver dopant is present as AgO (a weak line, −111 AgO) and as Ag^0^ (a weak line 38.1 degrees, plane 111). The composite film with the lowest Ag concentration (Sol C) is well crystallized mainly in anatase (with the crystallite size of 15.7 nm (101 line). No rutile fraction is recorded. The very broad weak line indicates AgO formation. The peak of Ag^0^ is clearer, but still too broad and weak. Probably, Ag^0^ NPs are amorphous and/or very tiny (under XRD detectable limits). Similar findings were previously reported by other researchers [27].

Figure 3d presents XRD patterns of TiO_2_:Ag films, annealed at 600 °C. TiO_2_:Ag (Sol A) film shows a polycrystalline multiple phase structure. The anatase phase is expressed with its strongest line, and the crystallite size is 10.8 nm. XRD lines appearing at 36.1°, 41.1°, and 54.1° are indexed as (101), (111), (211) planes of the rutile TiO_2_. The crystallite size of 26.7 nm (110) is determined. AgO fraction is also seen, and the crystallite size is 8.2 nm. Cubic Ag^0^ is expressed by the strong line (38.1 degrees, (111)) with crystallite size of 26.7 nm. The peaks at 13.1 degrees (200 plane) and at 64.4 degrees (220 plane) are also assigned to Ag^0^. No AgO phase for TiO_2_:Ag films, obtained from Sol B and Sol C, is detected. The silver incorporation induces the formation of Ag nanoparticles with the sizes of 12.6 (Sol B) and 23.2 (Sol C) nm. In the structure of TiO_2_:Ag film (Sol B), the co-existence of anatase and rutile phases is found, with the crystallite sizes of 14.0 and 31.4 nm, respectively. TiO_2_:Ag film, obtained from Sol C, crystallizes in the anatase titania without the traces of other TiO_2_ phases. The respective crystalline size is 16.3 nm (101).

**Figure 4:**
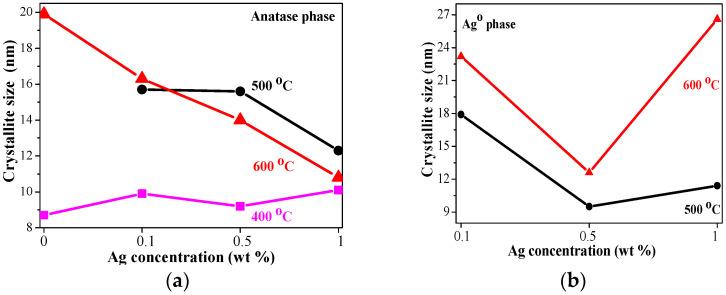
The dependence of the crystallite size on Ag concentration at different temperature treatments, where (**a**) presents the size of anatase phase and (**b**) the size of the metallic Ag NPs. The crystallite sizes are shown for samples, annealed at 400 °C (magenta line and squares), at 500 °C (black line and bullets) and at 600 °C (red line and triangles).

Figure 4 illustrates the effect of the Ag precursor concentration on the crystallite sizes of anatase phase and of Ag NPs as a function of the annealing temperature. After 400 °C treatment, the crystallites of anatase phase are grown bigger with increasing Ag doping. When the temperatures rise to 500 and 600 °C, it is found that the silver presence provokes the growth of smaller anatase crystallites, as their size gradually decreases with Ag addition. This can also be explained with the presence of AgO and different TiO_2_ crystal phases. Ag NPs (see Figure 4b) grew with the smallest size in the TiO_2_:Ag films, obtained from Sol B and annealed at 500 and 600 °C. The greatest Ag NPs are determined for Sol A nanocomposite film, treated at 600 °C. These results support the FESEM study.

**Figure 5:**
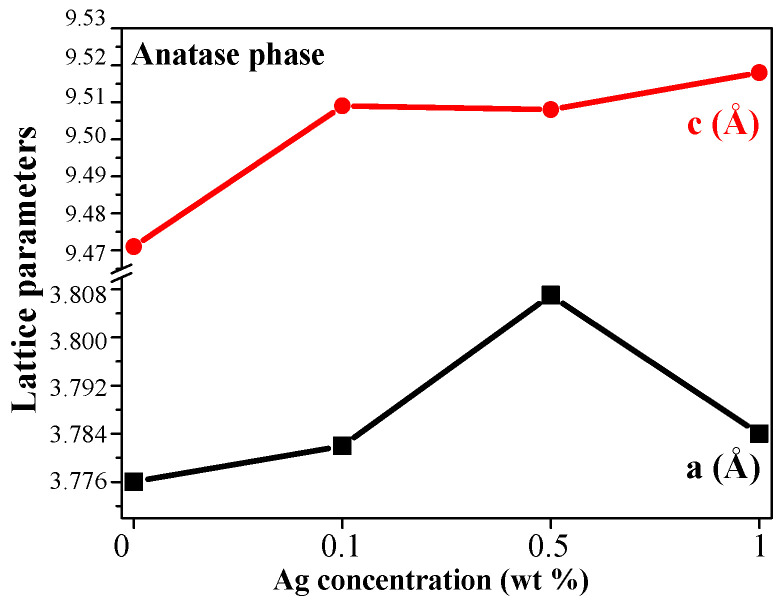
The lattice parameters of anatase phase in TiO_2_ and TiO_2_:Ag films, treated at 600 °C, where the red color and bullets present the c parameter and the black line and squares shows a parameter in dependence of the silver concentration.

The change in the lattice parameters of tetragonal anatase phase of TiO_2_ and TiO_2_:Ag films, treated at 600 °C as a function of Ag concentration, is shown in Figure 5 and Table 2. It is found that there is a lattice expansion with Ag doping, except for the slight decrease of the *a* axis for TiO_2_:Ag film, deposited from Sol A. The greater values of the lattice parameters can be explained by the successful dissolution of Ag within TiO_2_ host lattice by substitution of Ti sites, as the ionic radius of Ag^+^ (0.126 nm) is greater than that of Ti^4+^ (0.068 nm) [28].

XRD analysis shows that the AgNO_3_ initial concentration in the Ti sol solutions plays a significant effect on the film structure. Ag doping induces the appearance of rutile phase. Other authors [29] also found that Ag presence can promote the formation of rutile phase as it lowers the anatase to rutile transition temperature. On the other hand, the combined influences of the Ag concentration and the thermal treatment determines greater Ag NPs with the partially oxidation of silver at certain annealing temperatures.

#### 2.2.2. XRD Analysis—Effect of UV Treatment on the Sol Solution

**Figure 6:**
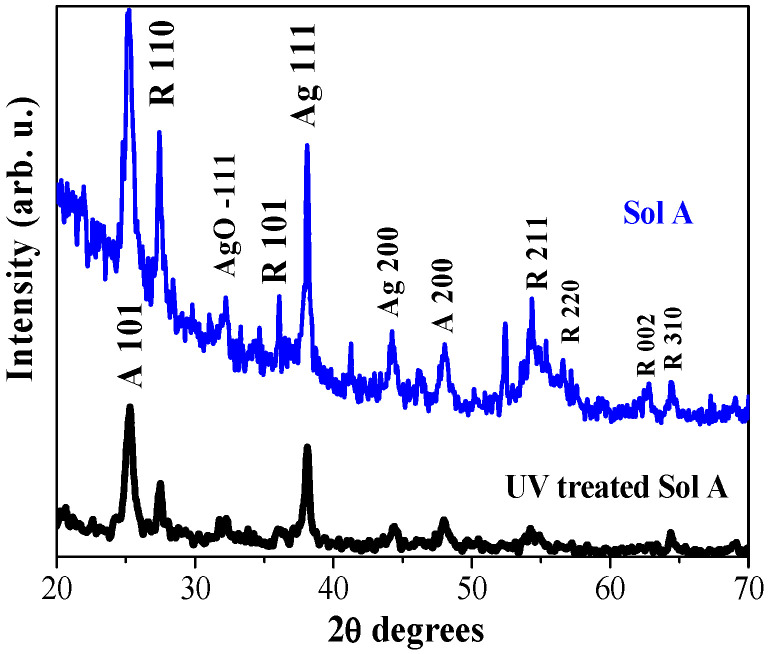
XRD patterns of TiO_2_:Ag films, deposited from Sol A (1% wt AgNO_3_) and from UV-radiated Sol A. The films are annealed at 600 °C. The anatase phase TiO_2_ is marked as A and rutile as R, respectively.

Figure 6 shows the comparison of XRD patterns of TiO_2_:Ag, obtained from Sol A and from preliminary UV-radiated Sol A for 2 h. XRD pattern of TiO_2_:Ag film, deposited from UV-treated Sol A, exhibits lower crystallization with intense anatase lines in contrast to TiO_2_:Ag (Sol A) film, where the rutile phase is manifested by more and stronger XRD features. AgO fraction is detected in the two sets of samples, but the film obtained from UV-treated Sol A reveals a broad XRD line with lower intensity. The films from Sol A with no preliminary treatment show AgO crystallites with a size of 8.2 nm. UV treatment of the sol before film deposition results in the low crystalline films.

#### 2.2.3. XRD Analysis—Anatase/Rutile Fraction in TiO_2_:Ag Films

**Figure 7:**
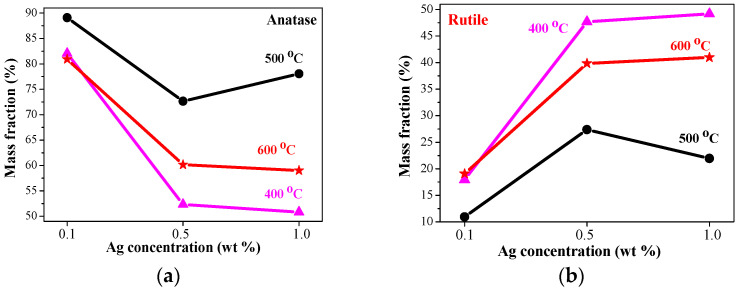
Crystallographic composition (weigth percentage %) of (**a**) anatase and (**b**) rutile phases of TiO_2_:Ag films as a function of Ag concentration (first group) depending on the annealing temperature 300–600 °C. The corresponding mass fractions are shown of samples, annealed at 400 °C (magenta line and squares), at 500 °C (black line and bullets) and at 600 °C (red line and asteriks).

In order to study the effect of the Ag amount and the technological procedures on the film crystallization, the anatase and the rutile contens in TiO_2_:Ag films are determined from formulas [30]:

(1) $$W_{A}=\frac{0.886I_{A}}{0.886I_{A}+I_{R}},$$

(2) $$W_{R}=\frac{I_{R}}{0.886I_{A}+I_{R}}$$

where *W_A_* and *W_R_* represent the mass fraction of anatase and rutile TiO_2_, respectively. *I_A_* and *I_R_* are the integral intensities of the anatase (101) and the rutile (110) peaks. The estimated values are shown in Figure 7.

The rutile phase is provoked by the Ag incorporation. Higher Ag doping promotes the higher rutile fraction. The rutile phase mass fraction depends greatly on the annealing temperatures. TiO_2_:Ag films, obtained from Sol C (smallest Ag concentration), possess the highest anatase content with small rutile inclusion from 10.9 to 19.1% (see Table 2). The anatase and the rutile mass fractions in TiO_2_:Ag films, deposited from Sol A treated at 600 °C, are 59.02 and 40.98 wt%, respectively, while the values for the films obtained from UV-radiated Sol A are 73.71 (anatase) and 26.29 wt% (rutile fraction), respectively, as can be seen from Table 2. The formation of the anatase and the rutile phases and the variation of their mass fractions with Ag doping concentration can be interpreted with the presence of Ag and AgO phases. Similar findings have been previously reported [31]. Generally, the silver doping promotes the rutile formation [18]. Ag presence can lead to decreasing of the anatase grain sizes and an increase in the surface defects, which enhances the rutile nucleation and growth [32]. Another effect is that the dopant can change oxygen vacancies. Ag incorporation is supposed to enhance oxygen vacancies, facilitating the structural rearrangement for the rutile crystal formation [32].

XRD study reveals that Ag NPs incorporation causes the modification of TiO_2_ film crystallization. New crystal phases appear, absent in the structure of the undoped samples. It is determined that the establishment of the rutile phase is strongly dependent on Ag doping concentrations and on the treatments applied. AgO fraction is vanished after annealings above 500 °C and it is almost absent for the lower silver concentrations in the case of the thermally treated TiO_2_:Ag films.

### 2.3. FTIR Analysis

**Figure 8:**
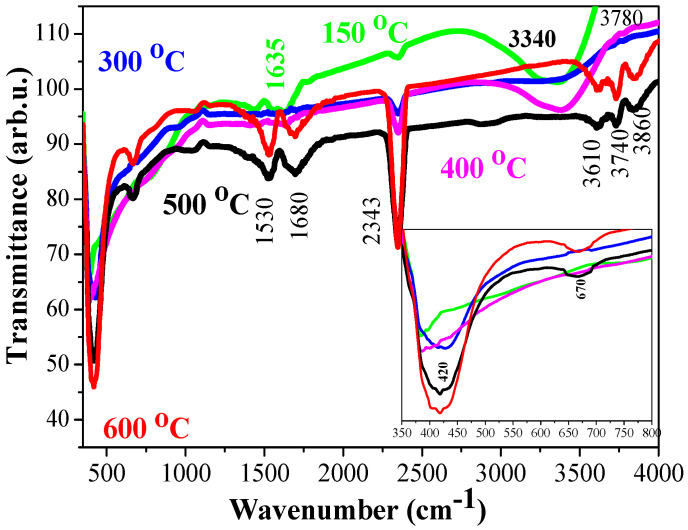
FTIR spectra of sol-gel pure TiO_2_ films, treated at 150 °C (preheated sample, green line), 300 °C (blue line), 400 °C (magenta line), 500 °C (black line) and 600 °C (red line), are shown, with the inserted figure presents the enlarged spectra in 350–800 cm^−1^.

FTIR spectra of sol-gel TiO_2_ films, annealed at temperatures 300–600 °C, are shown in Figure 8. It is seen the absorption bands and their positions and intensities change with the thermal treatments. The absorption bands related to OH stretching vibrations appear in the spectral range 3200–3800 cm^−1^. The preheated film (150 °C) manifests a very broad and strong band at 3340 cm^−1^, which is considered an overlapping of the stretching mode of interacting hydroxyl groups (involved in hydrogen bonding) and symmetric and antisymmetric modes of molecular water coordinated to Ti^4+^ cations [33].

Similar absorption bands with lower intensities are observed in the spectra of TiO_2_ films, annealed at 300 and 400 °C. The spectrum of 400 °C annealed TiO_2_ film reveals a new absorption feature at 3780 cm^−1^, due to surface OH^−^ groups [33,34]. With increasing the annealing temperatures, FTIR spectra show absorption bands at 3860 (hydroxyl surface groups), 3740 (free OH groups), and 3610 cm^−1^ (stretching non-hydrogen bonding and isolated hydroxyl group [35]). The corresponding bending OH vibrations are detected at 1610–1640 cm^−1^, clearly seen for the preheated sample (Figure 8, green line) [36]. After annealing, this band is shifted to 1680 cm^−1^. The broad band 1610–1690 cm^−1^ can be due to the contribution of the bending mode of OH groups and C=O stretching vibrations [37]. The IR line at 2343 cm^−1^ (seen in all spectra) is assigned to C–O vibrations of CO_2_ (atmospheric air) as the measurements are conducted in air [38]. The line at 2930 cm^−1^ is due to C–H bonds [39]. The lines at 1520–1540 cm^−1^ and the weak band at 1440 cm^−1^ are related to carboxyl C=O vibrations and methylene groups [39]. The line at 1100 cm^−1^ (FTIR spectra of TiO_2_, annealed at 300–600 °C) is to alkoxy C–O [36].

Characteristic IR absorptions due to metal–oxygen bonds are manifested below 1000 cm^−1^. The preheated sample shows a line at 766 cm^−1^, related to Ti–O stretching vibrations [39]. With increasing the annealing temperature, a line at 670 cm^−1^ is detected, attributed to Ti–O–O bonds [35]. The main absorption band becomes stronger with increasing the annealing temperature, as it shifts from 395 cm^−1^ (preheating) to 420 cm^−1^. This band is assigned to the stretching vibrations Ti–O in anatase TiO_2_ [38,40].

**Figure 9:**
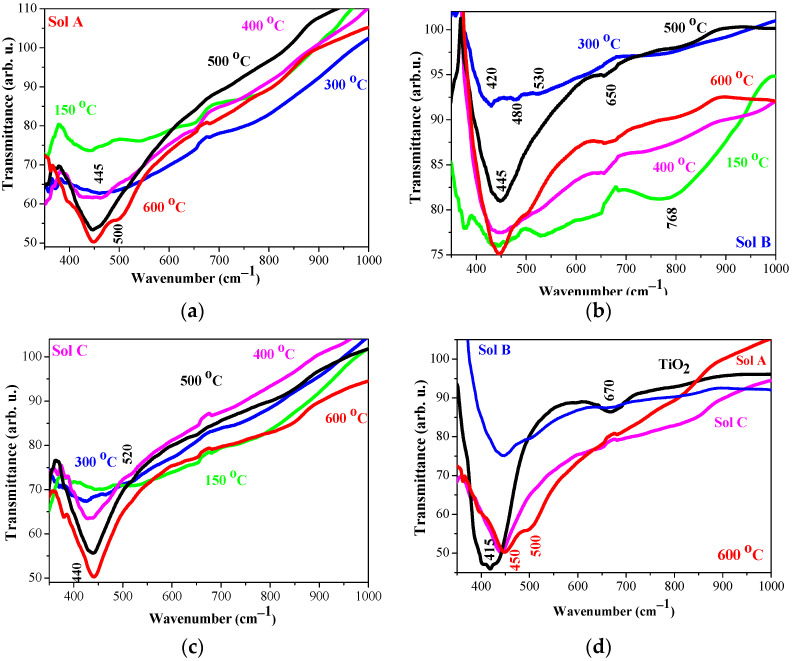
FTIR spectra of sol-gel TiO_2_:Ag films, treated at 300–600 °C, where (**a**) presents the films, deposited from sol A; (**b**) from Sol B; (**c**) from Sol C, and green lines show FTIR spectra of the preheated sample at 150 °C, blue lines of 300 °C treated films, magenta, black and red lines of the films, annealed at 400 °C, 500 °C and 600 °C, respectively. Figure (**d**) exhibits the comparison of FTIR spectra of TiO_2_ (black line) and Ag-doped TiO_2_ films, treated at 600 °C and obtained from Sol A (red line), Sol B (blue line) and Sol C (magenta line).

The silver incorporation in TiO_2_ matrix leads to changes in FTIR spectra of the corresponding nanocomposite films, as seen in Figure 9. Hydroxyl group stretching and bending vibrations can be observed only for preheated and partially for 300 °C annealed samples. After higher temperature treatment, absorption features due to hydroxyl groups disappear. For this reason, FTIR spectra are given in the spectral range 350–1000 cm^−1^. The main absorption bands of TiO_2_:Ag films are broader, indicating contributions of many metal–oxygen bondings. The broadening and the complicated shape of the main absorption bands is occurred from the multiphase nature of TiO_2_:Ag films and the presence of anatase and rutile TiO_2_, AgO, and Ag crystalline phases. The broad complex shape can also be due from disordered structure and surface defects [41]. TiO_2_:Ag films, obtained from Sol A, reveal broad main absorption bands that become more intense with increasing the annealing temperatures. The characteristic band for anatase TiO_2_ is located at 420–440 cm^−1^ [33,35] and for rutile phase at 480 cm^−1^ [42,43]. The broadness of the absorption bands is due to the contributions of different metal–oxygen vibrations, including those characteristic for Ag–O. IR peaks related to AgO are reported at 518 cm^−1^ and 446 cm^−1^ [44].

TiO_2_:Ag films (Sol B) also show broad main absorption band, suggesting presence of different oxide fraction. The line at 768 cm^−1^ (in the spectra of preheated, 300 and 400 °C films) is attributted to Ti–O–Ti vibrations. The clear band at 650 (all temperatures) and at 530 cm^−1^ (preheated) is due to Ti–O stretching mode or due to Ag–O [45]. TiO_2_:Ag films (deposited from Sol C) have more featureless spectra below 1000 cm^−1^. The main absorption bands are very broad and they become stronger after 400 °C annealing. The broadness of IR bands can be due to an amorphous, disordered film structure [41]. These suggestions are confirmed by XRD study of TiO_2_:Ag films, deposited from Sol C.

The comparison in Figure 9d clearly demonostrates the effect of Ag incoorpoaration in TiO_2_ lattice. The main IR band is shifting towards higher wavenumbers. A similar finding has been finding for Ag NPs in TiO_2_ nanocrystals [46]. The silver incorporation in TiO_2_ results in absorption bands that are broader and weaker in comparison of those of sol-gel TiO_2_ films. These bands show a clear shift from 415 to 450 cm^−1^ (illustrated in Figure 9d). The broadest absorption band is exhibited for TiO_2_:Ag (Sol A) films, with a clear shoulder at 510 cm^−1^. Some authors claim that there are the stretching vibrations of Ag–O ionic bond groups [47]. IR features due to rutile TiO_2_ can also contribute to these bands. The main absorption band of TiO_2_:Ag (Sol B) films shows also a possible overlapping with Ag–O vibrations. XRD study of TiO_2_:Ag films (deposited from sol A) found out the formation of AgO fraction in the film structure.

**Figure 10:**
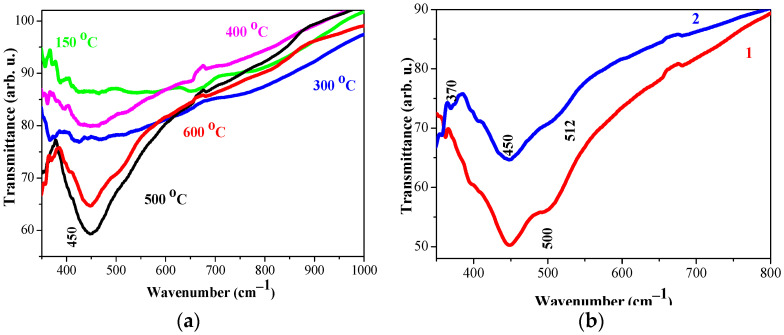
FTIR spectra of (**a**) TiO_2_:Ag films, deposited from UV-treated Sol A and annealed at different temperatures, where green line shows FTIR spectra of the preheated sample at 150 °C, blue lines of 300 °C treated films, magenta, black and red lines of the films, annealed at 400 °C, 500 °C and 600 °C, respectively and (**b**) the comparison of TiO_2_:Ag films, where curve 1, red line is the film obtained from Sol A and curve 2, blue line from UV-radiated Sol A. The films are annealed at 600 °C.

Figure 10a shows FTIR spectra recorded for TiO_2_:Ag films, deposited from the preliminary UV-treated Sol A (second group). The main bands are very broad, and new weak lines appear. The water inclusion diminishes with the higher temperature annealings. The main bands are positioned at 445–450 cm^−1^ for 400–600 °C treated films, as lower-temperature annealed films have no clear main line. Figure 10b manifests that UV radiation of the sol before thin film deposition has a slight impact on the vibrational properties of the nanocomposite TiO_2_:Ag films.

FTIR investigation reveals that the film structure is modified by Ag embedment, the silver concentration, and annealing temperatures. UV radiation on sol solution affects the vibrational properties. FTIR analysis confirms XRD conclusions.

### 2.4. Optical Properties of TiO_2_:Ag Films

#### 2.4.1. Optical Properties of Thermally Treated TiO_2_:Ag Films (First Group)

The influence of the technological parameters and the film treatments on the optical behaviour of TiO_2_:Ag films was characterized by UV–VIS spectroscopy. It is well known that UV–VIS spectroscopy is one of the most applied characterization methods to determine metal nanoparticles existence [48].

**Figure 11:**
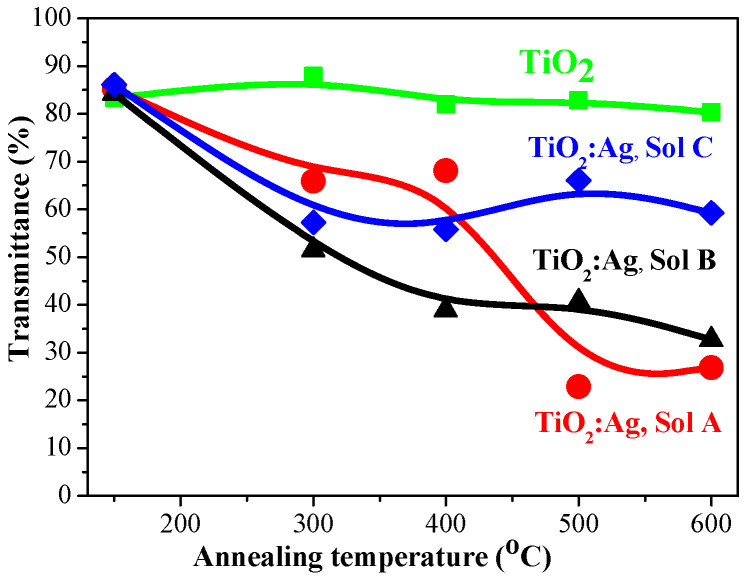
Comparison of the average transmittance values, determined for the spectral range 450–800 nm as a function of the annealing temperatures.

The change in the optical transmittance average values in dependence on the annealing temperatures of the first group samples is shown in Figure 11. All the samples possess high transparency (83–86%) after preheating procedure. The annealing at higher temperatures results in a decrease in transmittance.

Undoped TiO_2_ films retain their high transparency with a slight diminishing with annealing (from 87.9% (300 °C) to 80.3% (600 °C)). The nanocomposite TiO_2_:Ag films reveal a significant lowering of transmittance values after thermal treatments above 300 °C. TiO_2_:Ag films (Sol A), annealed at 300 and 400 °C, possess greater transmittance than films with lower Ag doping. This changes after the treatments at 500 and 600 °C, as the transmittance of TiO_2_:Ag films (Sol A) drops sharply from 85.4% to 26.9% (600 °C). TiO_2_:Ag films, obtained from Sol B, show a gradually decrease of the transmittance with the annealing temperatures. TiO_2_:Ag films with lowest Ag incorporation (Sol C) exhibit different optical behavior: the transmittance values are closed (55.8–59.2%) and a peak of 66.1% for the higher annealled film at 500 °C.

**Figure 12:**
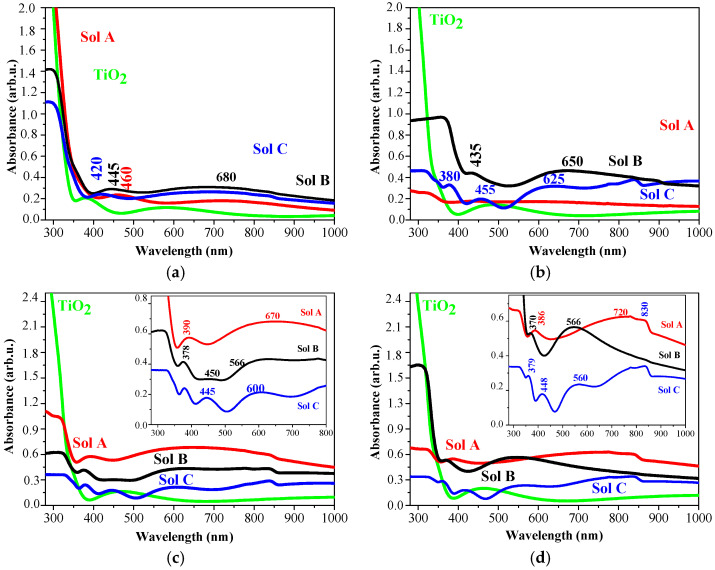
Absorbance spectra sol-gel TiO_2_ (green lines) and TiO_2_:Ag films, obtained from Sol A (red lines), Sol B (black lines), and Sol C (blue lines) as (**a**) 300, (**b**) 400, (**c**) 500, and (**d**) 600 °C (First group).

Figure 12 presents the absorbance spectra of undoped TiO_2_ and TiO_2_:Ag films, obtained from the sols with different Ag content and treated at 300, 400, 500, and 600 °C. The undoped TiO_2_ films possess very low absorbance in the visible spectral range, indicating high transparency. The spectra show high absorption below 360 nm, which can be attributed to the intrinsic TiO_2_ band gap transition from 2p orbitals of the oxide anions in the valence band to 3d orbitals of Ti^4+^ cations in the conduction band [49]. TiO_2_ films also show some absorption bands in the visible spectral region, possibly due to the existence of surface defects [50]. Increasing annealing temperature slightly induces absorbance.

The incorporation of Ag gives rise to higher optical absorbance in the whole visible spectral range, likely due surface plasma resonance. These observations are in agreement with other authors [51,52]. The presence of Ag nanoparticles in TiO_2_ enables the nanocomposite films to absorb in the visible region, thus leading to improvement of their efficiency in various applications such as optoelectronic devices and energy harvesting processes [53]. Plasmonic nanostructures can also enhance light scattering in thin-film solar cells, increasing the absorption in semiconductor absorber [54]. The optical absorbance intensity increases with higher silver concentration. TiO_2_:Ag films, obtained from Sol A, reveal the highest absorbance for the films annealed at 500 and 600 °C. Many factors alter the absorption of Ag NPs, such as NP sizes, shapes, dielectric constant of the matrix, and particle agglomeration [53]. The absorptions at 420–460 nm in TiO_2_:Ag films, annealed at 300–500 °C, are due to SPR of Ag NPs, related to dipole resonant wavelength of spherical Ag NPs [54]. For these nanocomposites films, it can be observed very broad absorption bands centered from 730 to 566 nm (depending on the Ag concentration). The broadness and red-shifting of absorption are associated with larger Ag NPs and the presence of higher-order resonances [55].

TiO_2_:Ag films, obtained from Sol A and annealed at 400 °C, shows an almost flat and featureless spectrum. Meanwhile, Sol C films present clear absorptions around 380 nm (assigned to formation and growth of isolated silver nanoparticles [56] or/and combined effects of the presence of anatase titania, Ag, and Ag oxide nanostructures [57]) and broad bands at 615–650 nm, related to SPR of non-spherical Ag NPs with larger size distribution and/or to higher order plasmon modes [57]. The TiO_2_:Ag film from sol C shows an additional band at 455 nm. After annealing at 500 °C, the absorbance spectra show interesting distinctions as TiO_2_:Ag film, deposited from Sol A, has the highest absorption in the visible spectral area and reveals the absorption bands at 386 and a broader band around 720 nm. Sol B films show the bands at 378 nm and around 600 nm. Films derived from Sol C reveal absorption bands at 379, 448, and 560 nm. These bands are due to SPR absorption of Ag NPs with asymmetric shapes [58] and/or LSPR of interconnected silver nanoparticles [59]. The absorption decreases with decreasing the Ag doping. The highest annealing temperature induces clear absorption at 370–389 nm in TiO_2_:Ag films.

It is found that the Ag NPs incorporated in TiO_2_ films promote strong absorption in visible spectral range. The position and broadness of LSPR are influenced by the silver concentrations (more silver nanoparticles can create smaller interparticle spacing), TiO_2_ crystal structure (anatase and rutile phases detected) and the annealing temperatures (300–600 °C). The broad absorption bands can be attributed to nonuniformity and asymmetrical shapes of Ag NPs, clusters, and agglomeration of metal nanoparticles, growth of larger particles, or oxide shell formation of metal nanoparticles. Similar effects are reported by many authors [59,60].

#### 2.4.2. Optical Characterization of the Synthesized Sol Solutions

**Figure 13:**
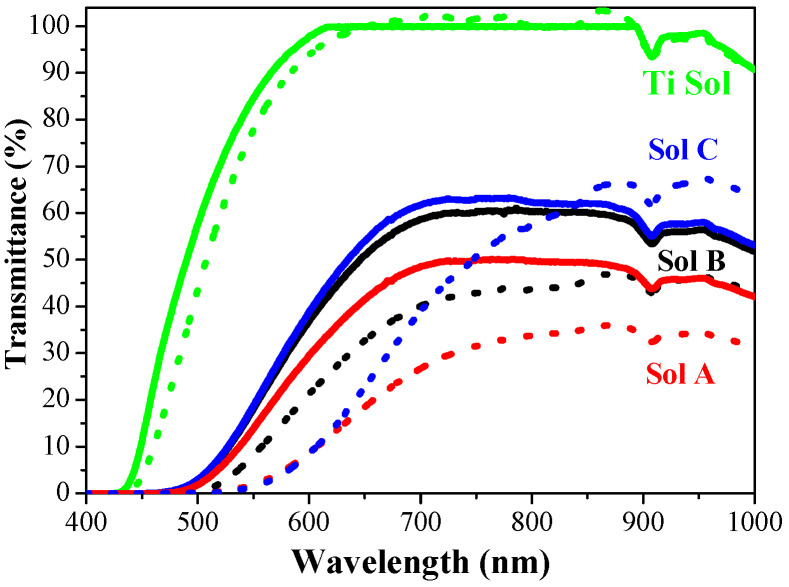
Transmittance spectra of the sol solutions for depositing TiO_2_ and TiO_2_:Ag films before (solid lines) and after (dot lines) UV treatment for 2 h.

The effect of UV radiation on the synthesized sol solutions is investigated. The transmittance spectra of the sol solutions for depositing TiO_2_-based films were measured before and after UV treatment. Figure 13 presents the transmittance spectra in the spectral range 400–1000 nm. It is clearly seen that the sol for TiO_2_ deposition is very transparent, with nearly 99% transmittance for wavelengths above 610 nm. The sols for obtaining TiO_2_:Ag films (Sols A, B and C) possess significantly lower transmittance. Their transparency in their initial state is reduced with increasing the silver concentration from 62% (above 700 nm, Sol C) to 50–45% (above 700 nm, Sol A). The absorption edges are located at 520 (Sol A), 500 (Sol B), and 497 (Sol C) nm, respectively.

UV light radiation, applied to the pure sol solution results in a slight transmittance change and the shifting of the absorption edge from 440 to 460 nm (see Figure 13, the green dot line). The effect of UV radiation on the sols, containing silver species is noticeable. It is observed that transparency is significantly reduced for Sol A and Sol B after UV treatment (Figure 13, red and black dot lines). Their absorption edges are red-shifted to 620 and 570 nm, respectively.

The sol solution prepared with the lowest silver concentration (Sol C) also reveals a great difference after UV treatment as its absorption edge shifts to 625 nm (blue lines). It is also found that the transparency of Sol C improves for wavelengths above 845 nm. The addition of AgNO_3_ to titanium sol solution changes the color of the solution from light yellow to deep brown, which can be an indication of the reduction of Ag^+^ to Ag° as well as the red shift of the absorption edges of Ag-containing sols to the visible-light region above 570 nm.

#### 2.4.3. Optical Properties TiO_2_:Ag Films, Obtained from UV Radiated Sols (Second Group)

**Figure 14:**
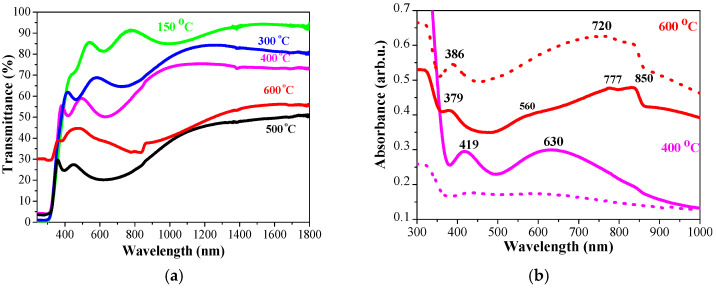
(**a**) Transmittance spectra of TiO_2_:Ag films, obtained from UV-treated Sol A in dependence of the annealing temperatures, where green line shows the spectrum of the preheated sample at 150 °C, blue line of 300 °C treated films, magenta, black and red lines of the films, annealed at 400 °C, 500 °C and 600 °C, respectively and (**b**) comparison of absorbance spectra of TiO_2_:Ag films, obtained from UV-treated Sol A (solid lines) and from untreated Sol A (dotted lines) after annealing at 400 and 500 °C.

The transmittance and absorbance spectra of TiO_2_:Ag films, obtained from UV-radiated Sol A, are shown in Figure 14. The comparison of the spectra of the films, obtained from untreated Sol A and UV-radiated Sol A, exhibits slight difference after annealing at 300 and 500 °C. Significant distinction can be observed for 400 and 600 °C treated TiO_2_:Ag films (Figure 14b). The absorbance spectrum of TiO_2_:Ag film (Sol A) at 400 °C is flat and featureless, in contrast to the spectrum of TiO_2_:Ag film, deposited from UV-radiated Sol A, where two maxima can be established: a clear peak at 419 nm and a broad absorption band at 630 nm. The absorption at 419 nm is related to the SPR effect of spherical Ag nanoparticles [61]. The appearance of the second wide absorption band at longer wavelengths (630 nm) can be explained by an increase of the Ag NPs sizes; consequently, interparticle distances become smaller, leading to aggregation or clustering [62]. The high temperature annealing at 600 °C results in significant higher absorption across visible range of TiO_2_:Ag film, deposited from untreated Sol A in comparison to the nanocomposite film obtained from UV-radiated Sol A.

The spectrum of TiO_2_:Ag film from UV-radiated Sol A exhibits two absorption bands at 379 nm (closed to the observed one for the other sample at 386 nm) and an asymmetric broad band centered at 730 nm with two additional maxima at 588 and 800 nm.

There is a similarity between the absorption band positions of 600 °C annealed TiO_2_:Ag films, derived from untreated and UV-radiated Sol A. The band at 683 nm (untreated Sol A film) is not observed in the other sample spectrum, despite a maximum at 588 nm is observed. This band can be assigned to plasmon resonance of bigger Ag NPs or two nanoparticles dimers [60,63].

#### 2.4.4. Optical Band Gap of TiO_2_:Ag Films

**Figure 15:**
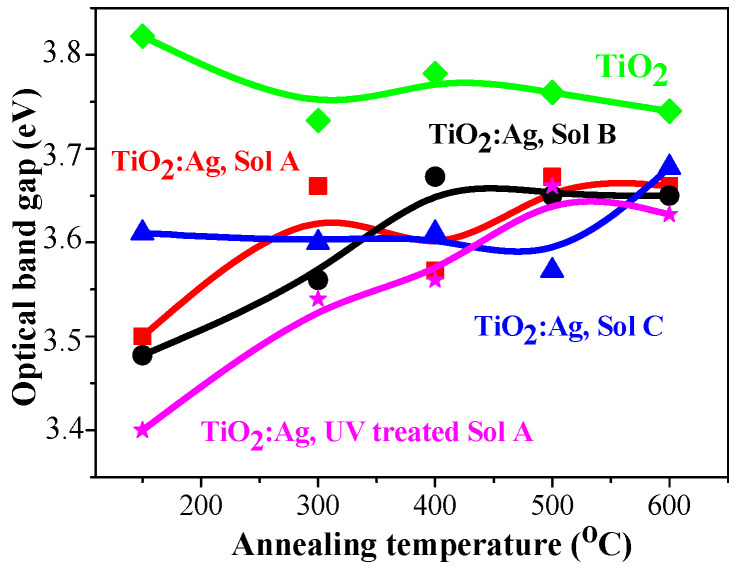
Optical band gap of TiO_2_ and TiO_2_:Ag films, depending on the annealing temperatures, where green line and squares shows thevalues of TiO_2_ films, red line and squares of TiO_2_:Ag films from Sol A, black line and bullets—Sol B samples, blue line and triangles—Sol C films and magenta line with asteriks—TiO_2_:Ag films, obtained from preliminary UV radiated Sol A.

Figure 15 shows the changes of the optical band gap of TiO_2_ and TiO_2_:Ag films as a function of the annealing temperatures. The determination of optical band gap (E_g_) values for thin films is of great importance in the materials science. In the case of TiO_2_, a smaller E_g_ allows more efficient absorption of solar energy, whereas a wider optical band gap is reported to be beneficial for application in electronic devices, optical coatings, and waveguides [64].

It is found that there is a little reduction of the optical band gap of sol-gel TiO_2_ films (from 3.82 (preheating film) to 3.74 eV (600 °C)). A similar narrowing with thermal treatments (3.69–3.79 eV) has been previously reported and is related to the improved crystallinity and greater crystallites [65].

In the case of Ag doping, the increase of the annealing temperature provokes a widening of the optical band gap, most clearly manifested for TiO_2_:Ag films, obtained from UV-treated Sol A. TiO_2_:Ag films, annealed at 600 °C, exhibit closed E_g_ values (in the range of 3.63–3.68 eV). The variation of the optical band gap can be related to the complicated crystalline film structure, as revealed by XRD study, which shows a multiphase film structure with the existence of anatase and rutile TiO_2_, Ag metallic, and AgO phases. Ag presence influences the Eg values, as the values of the nanocomposite TiO_2_:Ag films are significantly lower than those of pure TiO_2_ [66]. It was previously reported [67] similar effect in Ag-doped TiO_2_ NPs, attributed to Ag dopant migration into the TiO_2_ lattice, thus creating localized energy between the VB and CB, thereby lowering the band gap. These observations are consistent with previous research [66,67,68]. The obtained optical band gap values of TiO_2_ and TiO_2_:Ag films are in good agreement with the reported values in the literature for TiO_2_-based materials [69,70].

## 3. Materials and Methods

The sol solution synthesis for depositing TiO_2_ film has been previously described [26]. The precursor was titanium ethoxide (>97% purity, Fluka Chemie, Buchs, Germany). The acetate modification (an exothermic reaction) was provoked by introducing the glacial acetic acid (100%, Merck KgaA, Darmstadt, Germany). The small amount of water caused the hydrolysis and condensation reactions and gel formation. The sol solution was achieved by involving acetyl acetone (>98%, Sigma Aldrich Chemie, Buchs, Germany) as a peptizing agent and stabilizer. The solution turned transparent after aging for 1 week. The silver precursor was AgNO_3_ (silver nitrate, ≤100%, Merck KGaA Frankfurter). Experiments were conducted with three different concentrations: 1, 0.5, and 0.1% wt AgNO_3_. The concentration of AgNO_3_ was chosen in order to investigate the effect of the concentration on the properties of the final nanocomposite films. The first experiment was performed with the highest concentration (1% wt AgNO_3_). Based on the obtained results, we decided to investigate the concentration effect by adding 2 and 10 times smaller amounts of AgNO_3_. The obtained mixed sols for depositing nanocomposite TiO_2_:Ag films were defined as Sol A (1% wt AgNO_3_), Sol B (0.5% wt AgNO_3_), and Sol C (0.1% wt AgNO_3_), respectively. The silver-containing sol solutions were stirred with a magnetic stirrer (ARE, Velp Scientifica s.r.l., Usmate, Italy) for 1 h. Then, the mixed sols were put in an ultrasonic bath (ultrasonic cleaner, UST 2.8–100, Siel Ltd., Gabrovo, Bulgaria) at the temperature of 50 °C. The duration of this procedure was few hours per day over a week. The final synthesized sols were stored in the dark bottles.

Since changes in size, shape, and distribution of Ag nanoparticles are fundamental parameters for modifying the plasmonic properties, two sets samples were fabricated. The first group was TiO_2_:Ag thin films with 5 layers from the three mixed sols. The second group of samples was deposited from the Sol A (1% wt AgNO_3_), which was pretreated with UV radiation for 2 h.

The thin films were obtained by spin-coating method (using Spin coater P 6708, PI-KEM Limited, Staffordshire, UK) on silicon wafers (FZ, p-type, resistivity 4.5–7.5 Ω, orientation <100>, diameter 25.4 mm) and quartz substrates (UV graded, glass thickness 1 mm ± 0.1). The quartz substrates were used for optical characterizationdue to their high transmission in both visible and UV light. On the other hand, Si substrates, which are transparent in the IR range, were applied to study the vibrational properties of TiO_2_-based films by FTIR spectroscopy in transmission mode.

The substrates were rotated at 8000 rpm/30 s after dropping a certain amount of the sols. The preheating temperature (drying between layers) was 150 °C for 30 min. The high temperature annealing procedures were at 300, 400, 500, and 600 °C for 1 h in air. The preheating and annealing treatments were performed in the tube furnace (Carbolite, Inc., Sheffield, UK) with with controllable heating and cooling rate of 10 °C/min.

The film thickness was determined using a LEF 3 M laser ellipsometer (manufactured by Siberian Branch of Russian Academy of Sciences, Novosibirsk, Russia), equipped with a HeNe laser at a wavelength of 638.2 nm. It was found that sol-gel TiO_2_ films possessed the film thickness from 270 nm (preheating sample) to 200 nm (600 °C). The silver-doped TiO_2_ films revealed greater values for film thickness: the films, obtained from Sol A (1% wt AgNO_3_), varied from 300 nm (150 °C) to 250 nm (600 °C). The nanocomposite films, deposited from Sol B (0.5% wt AgNO_3_), showed the film thickness in the range 285–230 nm. TiO_2_:Ag films from Sol C (the lowest AgNO_3_ concentration, 0.1% wt AgNO_3_) were 275–210 nm thick. The film thickness of the sol-gel produced films decreased with higher annealing temperature due to shrinkage and densification induced by the annealing treatments.

The film morphology was investigated by FESEM technique (Philips XL 30FEG-ESEM, FEI, York Probe Sources Ltd., York, UK). The investigated samples were coated with the gold film before the microscopic observation. Various magnifications were applied. X-ray diffraction patterns were recorded by the XRD diffractometer Bruker D8 (Bruker AXS GmbH, Karlsruhe, Germany) using Cu anode (λ_Kα_ = 1.54056 Å), at a grazing angle 2°, with a step time 8 s. FTIR spectra were taken with a Shimadzu Spectrophotometer IRPrestige-21 (with resolution of 4 cm^−1^, Shimadzu Corporation, Kyoto, Japan) over the spectral range 350–4000 cm^−1^, using a bare Si wafer as background.

The optical transmittance spectra were carried out by an UV–VIS–NIR Shimadzu 3600 double-beam spectrophotometer (with resolution of 0.1 nm, Shimadzu Corporation, Kyoto, Japan). The transmittance was measured against air. The average transmittance values were determined for the spectral range 450–800 nm. For measuring the transmittance spectra of the sol solutions, the solutions were placed in quartz cuvettes, with a bare quartz cuvette applied as reference.

Tauc relation was applied to estimate the optical band gap of the sol-gel films [71]:

(*αhν*) = *B* (*hν* − *E_g_*)^m^ (3)

where ‘*B*’ is a constant, *h* is Plank’s constant, *ν* is photon frequency, α is absorption coefficient, and *E_g_* is optical band gap. The constant *m* is the power factor characterizing the nature of electronic transitions between the valence band and the conduction band. It can take the following values: 1/2 for allowed direct, 2 for allowed indirect, 3 for forbidden direct, and 3/2 for forbidden indirect optical transitions.

The absorption coefficient, α is evaluated from the equation [72]:

(4) $$\alpha =-\frac{1}{d}In\left(\frac{{\left(1-R\right)}^{2}}{T}\right)$$

where *d* is film thickness, *T*—film transmittance, and *R*—reflectance, respectively.

Considering that TiO_2_ is a direct semiconductor [65], direct band gap values are determined by plotting (α*h*ν)^2^ vs. *h*ν curves and extrapolating the linear portion of the graphs to the energy axis, respectively.

## 4. Conclusions

Nanocomposite thin films of TiO_2_ films with embedded Ag nanoparticles were deposited by a facile and suitable sol-gel method. The impact of the silver dopant concentration (1, 0.5, and 0.1% wt AgNO_3_ in Ti sol solution) and thermal treatments (in the range of 150–600 °C) was investigated on the structural, morphological, and optical properties of the nanocomposite films. Undoped TiO_2_ films possess very smooth and uniform surface morphology. FESEM study reveals that the silver incorporation into TiO_2_ matrix modifies the film morphology. TiO_2_:Ag films with the highest Ag doping (Sol A, 1% wt AgNO_3_ in Ti sol solution) manifests rather rough morphology with tightly connected small spherical particles and large grains with the irregular shapes. The silver dopant in TiO_2_ also affects on the film crystallization behaviour. XRD analysis reveals the formation of the rutile phase at lower annealing temperatures. The change in anatase/rutile proportion is explored in dependence on Ag doping concentration and annealing temperatures. Ag incorporation promotes the rutile formation.

It is established that Ag is embodied in TiO_2_ films mostly as metallic Ag (as Ag NPs), but the presence and growth of AgO is also determined. The fraction of the silver oxide, AgO, is strongly influenced by the Ag concentrations in the sol solutions and is primarily found in samples annealed at the temperatures below 500 °C. For nanocomposite films derived from Sol C (0.1% wt AgNO_3_ in Ti sol solution), the AgO phase is almost absent. FTIR spectroscopy proves that silver incorporation modifies the shapes and the intensities of the absorption bands, revealing film disorder and rearrangement. FTIR analysis confirms XRD conclusions.

Optical characterization exposes the increased optical absorption across the visible range for TiO_2_ films with incorporated Ag NPs, attributed to the surface plasmon resonance effect. The optical absorption features suggest the growth of isolated Ag NPs, the formation of larger nanoparticles with irregular shapes, or/and the evolution of higher order resonance modes. These absorption bands depend strongly on UV and thermal treatments. Ag doping provokes a narrowing of the optical band gap (E_g_) in TiO_2_ films. The fabricated Ag–TiO_2_ nanocomposites offer promising applications in lasers, solar cells, and environmental remediation.

## Figures and Tables

**Table 1:** The summarized data for the crystallite sizes of the different phases in the sol-gel TiO_2_ nanocomposite films.

Annealing Temperature (°C)	Sample	Crystallite Size (nm) *			
		Anatase **	Rutile ***	Ag^0^ ****	AgO *****
300	TiO_2_	6.5 (6)	-	-	-
	TiO_2_:Ag (Sol A)	Broad, weak	3.7 (8)	Broad, weak	20.6 (5)
	TiO_2_:Ag (Sol B)	Predominantly amorphous			
	TiO_2_:Ag (Sol C)	Predominantly amorphous			
400	TiO_2_	8.7 (6)	No lines	-	-
	TiO_2_:Ag (Sol A)	10.1	20.5 (6)	21.8 (7)	24.9 (8)
	TiO_2_:Ag (Sol B)	9.2	19.3 (2)	broad	23.9 (7)
	TiO_2_:Ag (Sol C)	9.9	No lines	34.7 (6)	No lines
500	TiO_2_				
	TiO_2_:Ag (Sol A)	12 (1)	Broad, weak	11.4	Broad, weak
	TiO_2_:Ag (Sol B)	15.6 (7)	Broad, weak	9.5	24.1 (2)
	TiO_2_:Ag (Sol C)	15.7 (8)	No lines	17.9	Broad, weak
600	TiO_2_	20.0 (1)	-	-	-
	TiO_2_:Ag (Sol A)	10.8 (7)	26.7 (6)	26.6 (7)	8.2 (2)
	TiO_2_:Ag (UV Sol A)	13.4 (3)	27.0 (2)	22.7 (6)	10.9 (8)
	TiO_2_:Ag (Sol B)	14.0 (2)	31.5 (6)	12.6 (6)	No lines
	TiO_2_:Ag (Sol C)	16.3 (5)	No lines	23.2 (3)	No lines

* Estimated from ** XRD line 101 (anatase); *** XRD line 110 (rutile); **** XRD line (111) (metallic Ag), cubic Ag; ***** XRD line (−111) (AgO). The uncertainties in the cell parameters and the crystallite sizes are estimated by assuming a 0.02° uncertainty in the peak positions and in the FWHM.

**Table 2:** The lattice parameters and the anatase and the rutile contens for TiO_2_:Ag films, annealed at 600 °C.

Sample	a (Å)	c (Å)	W_A_ (%)	W_R_ (%)
TiO_2_	3.776 (3)	9.471 (6)	100	0
TiO_2_:Ag, Sol A 1 wt% AgNO_3_	3.784 (3)	9.518 (6)	59.0	41.0
TiO_2_:Ag, Sol A, UV treated sol	3.820 (3)	9.443 (6)	73.7	26.3
TiO_2_:Ag, Sol B 0.5 wt% AgNO_3_	3.807 (3)	9.508 (6)	60.2	39.8
TiO_2_:Ag, Sol C 0.1 wt% AgNO_3_	3.782 (3)	9.509 (6)	80.9	19.1

The uncertainties in the cell parameters and the crystallite sizes are estimated by assuming a 0.02° uncertainty in the peak positions and in the FWHM.

## Data Availability

Data are contained within this article.
